# Mephedrone (4-Methylmethcathinone): Acute Behavioral Effects, Hyperthermic, and Pharmacokinetic Profile in Rats

**DOI:** 10.3389/fpsyt.2017.00306

**Published:** 2018-01-10

**Authors:** Klára Šíchová, Nikola Pinterová, Monika Židková, Rachel R. Horsley, Eva Lhotková, Kristýna Štefková, Čestmír Vejmola, Libor Uttl, Marie Balíková, Martin Kuchař, Tomáš Páleníček

**Affiliations:** ^1^Department of Experimental Neurobiology, National Institute of Mental Health, Klecany, Czech Republic; ^2^Third Faculty of Medicine, Charles University in Prague, Prague, Czech Republic; ^3^Institute of Forensic Medicine and Toxicology, First Faculty of Medicine, Charles University in Prague, Prague, Czech Republic; ^4^Department of Physiology, Faculty of Science, Charles University, Prague, Czech Republic; ^5^Forensic Laboratory of Biologically Active Compounds, Department of Chemistry of Natural Compounds, University of Chemistry and Technology Prague, Prague, Czech Republic

**Keywords:** mephedrone, 4-methylmethcathinone, nor-mephedrone, pharmacokinetics, open field, prepulse inhibition, thermoregulation, Wistar rat

## Abstract

Mephedrone (MEPH) is a synthetic cathinone derivative with effects that mimic MDMA and/or cocaine. Our study in male Wistar rats provides detailed investigations of MEPH’s and its primary metabolite nor-mephedrone’s (nor-MEPH) pharmacokinetics and bio-distribution to four different substrates (serum, brain, lungs, and liver), as well as comparative analysis of their effects on locomotion [open field test (OFT)] and sensorimotor gating [prepulse inhibition of acoustic startle reaction (PPI ASR)]. Furthermore, in order to mimic the crowded condition where MEPH is typically taken (e.g., clubs), the acute effect of MEPH on thermoregulation in singly- and group-housed rats was evaluated. Pharmacokinetics of MEPH and nor-MEPH after MEPH (5 mg/kg, sc.) were analyzed over 8 h using liquid chromatography with mass spectrometry. MEPH (2.5, 5, or 20 mg/kg, sc.) and nor-MEPH (5 mg/kg, sc.) were administered 5 or 40 min before the behavioral testing in the OFT and PPI ASR; locomotion and its spatial distribution, ASR, habituation and PPI itself were quantified. The effect of MEPH on rectal temperature was measured after 5 and 20 mg/kg, sc. Both MEPH and nor-MEPH were detected in all substrates, with the highest levels detected in lungs. Mean brain: serum ratios were 1:1.19 (MEPH) and 1:1.91 (nor-MEPH), maximum concentrations were observed at 30 min; at 2 and 4 h after administration, nor-MEPH concentrations were higher compared to the parent drug. While neither of the drugs disrupted PPI, both increased locomotion and affected its spatial distribution. The effects of MEPH were dose dependent, rapid, and short-lasting, and the intensity of locomotor stimulant effects was comparable between MEPH and nor-MEPH. Despite the disappearance of behavioral effects within 40 min after administration, MEPH induced rectal temperature elevations that persisted for 3 h even in singly housed rats. To conclude, we observed a robust, short-lasting, and most likely synergistic stimulatory effect of both drugs which corresponded to brain pharmacokinetics. The dissociation between the duration of behavioral and hyperthermic effects is indicative of the possible contribution of nor-MEPH or other biologically active metabolites. This temporal dissociation may be related to the risk of prolonged somatic toxicity when stimulatory effects are no longer present.

## Introduction

Mephedrone (4-methylmethcathinone, 4-MMC; MEPH, hereafter), a synthetic derivative of cathinone was first synthetized in 1929 with the aim of developing this compound for therapeutic purposes (1). At the turn of the twenty-first century MEPH was rediscovered by recreational users (as a so-called “new psychoactive substance”: NPS) and owing to its psychoactive effects, it became widely used as party drug known under the street name “meow meow” (2, 3). Based on users’ reports, MEPH’s effects are very similar to amphetamine, to 3,4-methylenedioxymethamphetamine (MDMA) and to cocaine, or their combination (4–6). MEPH’s effects are rapid and of relatively short duration depending on the administration route (intranasal: ~30 min, oral: ~2–3 h) (7, 8), resulting in a tendency for recreational users to re-dose, as is the case with cocaine (9, 10). Prolonged and/or poly-drug use [including “slamming”—intravenous injection of MEPH combined with other drugs (11)] may be associated with adverse psychological (e.g., paranoia, depression, panic attacks), cardiovascular, or renal effects (12, 13). Furthermore, at least 90 deaths have been documented where MEPH alone (or its combination with other psychoactive compounds) was implicated (14–17). In 2010, MEPH was classified as a controlled substance in some European countries, and 2 years later in the USA (7). Despite its ban, it has remained a popular recreational drug to this day (18, 19).

Mephedrone acts as non-selective monoamine uptake inhibitor and releaser with dopamine transporter: serotonin transporter (DAT: SERT) inhibition ratio being 1.4, which led authors to label MEPH as mixed MDMA-cocaine-like compound (20, 21). However, while MEPH’s uptake of dopamine (DA) is roughly equivalent to that of serotonin (5-HT), it is (such as MDMA or cathinone) several times more potent at nor-epinephrine transporter (NET) with NET: DAT ratio being approximately 13 (20). MEPH is also active on vesicular monoamine transporters 2, where its activity is approximately 10 times less potent than MDMA (22). Partly contrasting the transporter studies, according to *in vivo* microdialysis studies in nucleus accumbens (NAcc), MEPH had approximately twofold greater effect on 5-HT than DA release (23, 24). Furthermore, MEPH also has some activity at serotonin 5-HT_2A_, noradrenaline α_1,2_ and trace amine associated receptor (TAAR_1_). Affinity for DAT together with its high blood–brain barrier permeability (twofold greater than amphetamine and MDMA) (20) and direct effects on DA in NAcc make MEPH a compound with high addictive potential, which is confirmed by users (10, 20, 25, 26) and by animal studies (27–29). Its strong affinity for NET then might be indicative of cardiovascular toxicity (7).

Mayer et al. (30), using *in vitro* assays, showed that the phase I metabolites 4-methylcathinone (nor-mephedrone (nor-MEPH) hereafter), 4-hydroxytolylmephedrone (4-OH-MEPH) and dihydromephedrone also have measureable activity at DAT, NET, and SERT, although of these, only nor-MEPH and 4-OH-MEPH at a range meaningful for behavioral tests. Therefore, bioactive metabolites can also contribute to MEPH’s effects. However, this was previously confirmed only for nor-MEPH, which displayed *in vivo* behavioral stimulatory activity (30).

In rodent models, MEPH administration leads to dose-dependent increases in locomotion [reviewed in Ref. (7)]. The intensity and duration of these changes is comparable to those observed after the same dose of MDMA, but lesser than amphetamine’s effects (23, 24). MEPH’s effect on sensorimotor gating has only been evaluated in a chronic administration paradigm by Shortall et al. (31); in order to mimic weekend type recreational use of drugs, they administered MEPH (1, 4, or 10 mg/kg) twice a week on two consecutive days for 3 weeks and tested prepulse inhibition of acoustic startle reaction [PPI ASR; a behavioral operationalization of sensorimotor gating (32)]; 30 min (min) after the final injection; this yielded no disruptive effect. On the other hand, related drugs, such as MDMA, amphetamine, cocaine, also cathinone itself, and methylone, have shown some disruptive effects in this paradigm (33–39). No information currently exists on MEPH’s acute effect nor the effects of its metabolites on PPI.

Studies of MEPH effects on thermoregulation are inconsistent in their results; both hyperthermic (Sprague-Dawley rats (24, 27)) and hypothermic (40) responses have been documented. Alteration of body temperature is an effect that is dose- and environment-dependent in the case of MDMA and related compounds [e.g., Ref. (38, 39, 41, 42)]. In two of our previous studies, we have found that serotonergic compounds, along with severe hyperthermia, can induce profound sweating, particularly when rats are housed in cages in groups (38, 41). Group-housing mimics the crowded conditions in clubs where drugs, such as MDMA and MEPH are typically used. It is generally known that the hyperthermia associated with the use of these compounds is one of the key preceding conditions of neurotoxicity as well as of acute somatic toxicity related to serotonin syndrome (43). Therefore detailed examination of dose-related interactions with environmental conditions (such as crowding) is necessary in order to elucidate inconsistencies in MEPH’s effects on thermoregulation.

Our main intention was to enrich current knowledge of MEPH by detailed description of the temporal characteristics of its behavioral effects in relation to its pharmacokinetics and bio-distribution and to investigate effects of its major active metabolite nor-MEPH. To describe the temporal profile of behavioral changes, two testing-onsets (5 or 40 min after drug administration) were used to register both peak and prolonged drug effects. Stimulatory locomotor effects, exploration and/or anxiogenic/anxiolytic potential were tested in the open field test (OFT) and the effects on sensorimotor gating were measured in PPI ASR. Alongside this, pharmacokinetic profile of MEPH and nor-MEPH in brain and serum, and their bio-distribution to liver and lungs were established, over 8 h. To evaluate MEPH’s effects on thermoregulation under crowded and isolated environmental conditions, rectal temperatures were measured over 8 h in groups of five rats versus rats housed alone.

## Materials and Methods

### Animals

Male outbred Wistar rats (VELAZ, Czech Republic) weighing approximately 180–250 g were housed in pairs under controlled conditions (light/dark arrangement: 12/12 hours, temperature: 22 ± 2°C, humidity: 30–70%) with *ad libitum* water and standard diet. In each study, rats acclimatized to the laboratory facility for seven days, with tests performed in the seven days following. Therefore, testing/sampling occurred when rats were approximately 10–11 weeks old (adult) and they were in the laboratory for approximately 10–14 days in total. During the acclimatization period, rats were handled four times and weighed twice. Experiments and measurements were conducted in the light phase of the cycle (between 07:00 and 15:00 h). Experimental groups consisted of 10 individuals, each rat was tested only once, with the exception that to reduce the number of animals used, rats treated by MEPH/nor-MEPH in behavioral studies were subsequently used for pharmacokinetic sampling. Hence, only eight additional rats were needed (for 30 min post-drug administration samples).

### Drugs and Chemicals

Mephedrone was purchased *via* the internet and subsequently purified and converted to MEPH hydrochloride by Alfarma s.r.o. (Czech Republic). The resulting MEPH was certified to be of 99.18% purity (analyzed by infrared spectroscopy) and also served as a reference standard for pharmacokinetic analyses using liquid chromatography. Nor-MEPH was synthesized at the Department of Organic Chemistry, Faculty of Chemical Technology (University of Chemistry and Technology Prague, Czech Republic) at a purity of 99.18%. Internal standards MEPH-D7.HCl and nor-MEPH-D7.HCl for quantitative liquid chromatography/mass spectrometry (LC/MS) assays were synthesized at the Department of Organic Chemistry, Faculty of Chemical Technology (University of Chemistry and Technology Prague, Czech Republic). Extraction columns (Bond Elut Certify 50 mg/3 ml) were supplied by Labicom s.r.o., Olomouc. Other chemicals used for laboratory purposes were of analytical grade purity. MEPH was stored in dry and dark place and dissolved in physiological saline (0.9% NaCl) immediately before experiments.

### Dosage

The doses for subcutaneous (sc.) administration were estimated with respect to the amounts usually used by humans, reported potency/affinity at transporters and based on our previous studies with related compounds especially MDMA, MDAI, and related ring-substituted cathinone methylone (35, 38, 39, 44, 45). Furthermore, we set these doses with the intention to mimic the dosage comparable to human use and intermediate—high dose with expected strong acute effect, but non-lethal toxicity. Finally, the doses were also adequately adjusted for interspecies differences according the formula suggested by Reagan-Shaw et al. (46). All substances were dissolved in vehicle (0.9% physiological saline) at a volume of 2 ml/kg administered sc. (for comparability with our previous studies). Rats used for pharmacokinetic sampling were treated by MEPH 5 mg/kg. MEPH 5 or 20 mg/kg was used in the temperature monitoring study, and MEPH 2.5, 5, or 20 mg/kg and nor-MEPH 5 mg/kg were used in behavioral tests. As vehicle controls (VEH) animals were treated with an equivalent volume of 0.9% physiological saline.

### Pharmacokinetics

For pharmacokinetics, rats were administered MEPH (5 mg/kg sc.) and subsequently decapitated after 30, 60, 120, 240, or 480 min (*n* = 8/experimental group). Sera, brain, liver, and lung tissues were collected and stored at −20°C until analysis.

#### Determination of MEPH and Nor-MEPH Levels in Serum and Tissue Samples Using LC/HRMS

##### Serum Pretreatment

0.2 ml of rat serum was fortified with the internal standard MEPH-D7 and nor-MEPH-D7 in methanolic solution (in an amount with respect to the levels of MEPH/nor-MEPH in assayed samples) and 0.5 ml of a 0.1 M phosphate buffer (pH 6) in a labeled tube.

##### Tissue Pretreatment

250 mg of tissue (brain, lung, liver) was homogenized with 5 ml methanol and the internal standard MEPH-D7 and nor-MEPH-D7 (in an amount with respect to the MEPH/nor-MEPH levels in samples). Each specimen was then ultrasonicated for 20 min and after supernatant separation by centrifugation, the supernatant was transferred into a clean labeled tube and evaporated to dryness. The residue was reconstituted in 0.1 M phosphate buffer (pH 6). For solid-phase extraction (SPE) of MEPH/nor-MEPH, a pretreated sample of serum or tissue, along with the buffer and internal standard, was loaded onto a Bond Elut Certify cartridge previously conditioned with 0.5 ml of 0.1 M phosphate buffer (pH 6). After application of each pretreated sample, the cartridge was washed with 0.5 ml of distilled water, 0.5 ml of 0.1 M HCl and 0.5 ml of CH_3_OH/H_2_O (1/1, v/v) and then air-dried for 5 min. The analytes were eluted three times with 0.5 ml of a freshly prepared mixture of dichloromethane/2-propanol/ammonium hydroxide (25%), 80/20/4, v/v/v. The eluate was gently evaporated to dryness under a stream of air at 40°C and then dissolved into mobile phase for LC/HRMS analysis.

#### LC/HRMS Conditions

The analyses were performed using Dionex Ultimate 3000 UHPLC coupled to an Exactive Plus-Orbitrap MS (ThermoFisher Scientific, Bremen, Germany) equipped with a HESI-II source. The chromatographic analyses of the serum and tissue samples were performed using a Kinetex PFP 100 A (50 × 2.1 mm, 2.6 mm) and Security Guard Cartridge PFP 4 × 2.0 mm (Phenomenex) with a flow rate of 400 ml/min, and gradient elution with 10 mM ammonium formate in 0.1% of formic acid as the mobile phase B. Gradient 0 min 5%, 4 min 45% B, 5–6 min held at 95%. The MS conditions were as follows: full MS in scan range of 50–500 *m/z* with positive electrospray ionization, resolution of 70000 FWHM (full width at half-maximum, scan speed 3 Hz), spray voltage of 3 kV, and an ion transfer capillary temperature of 320°C.

### Behavior: Open Field and PPI

#### Open Field

The OFT was performed in accordance with our previous studies (38, 47). An empty black square arena (68 cm × 68 cm × 30 cm) was used, which was virtually divided into a 5 × 5 grid of identical squares; 16 squares were located near the arena walls (comprising the peripheral zone), and 9 squares were situated centrally (comprising the central zone). Rats were placed individually into the center of the arena 5 or 40 min after the drug administration (testing-onset) and their behavior was recorded for 30 min (nor-MEPH-treated rats were tested at the 5 min testing-onset only). The software EthoVision Color Pro v. 3.1.1 (Noldus, Netherlands) was used to capture the raw data used in the calculation of the following dependent variables: trajectory length (cm; corrected for deviations of <3 cm) and its temporal dynamics in 5 min intervals; thigmotaxis (∑*f*_peripheral zones_/∑*f*_all zones_, where *f* = frequency of appearance in the zone) reflects the probability of appearance in the peripheral zone; *T*_center_ reflects time spent centrally (∑time_centralzones_).

#### Prepulse Inhibition

Prepulse inhibition was evaluated in two identical startle chambers (SR-LAB, San Diego Instruments, CA, USA) each consisting of a sound-proof, evenly lit, ventilated enclosure with a Plexiglas stabilimeter (8.7 cm inner diameter). The experimental design was adopted from our previous studies [e.g., Ref. (38, 41, 47)]. Briefly, 2 days before testing, rats were acclimatized to the startle chamber with a drug-free 5 min pre-training procedure consisting of 5 pulse alone stimuli (115 dB/20 ms) presented over background white noise (75 dB). Startle data were not recorded for acclimatization. On the test day, the testing session was initiated 5 or 40 min after drug administration (only 5 min for nor-MEPH). The test session consisted of 72 trials in total with an inter-trial interval (ITI) of 4–20 s (mean ITI: 12.27 s). After 5 min exposure to a continuous 75 dB background white noise, six 125 dB/40 ms duration pulse alone trials were delivered to establish baseline ASR (for later calculation of habituation). Following this, 60 trials of the following were presented in a pseudorandom order: (A) pulse alone: 40 ms/125 dB; (B) prepulse–pulse: 20 ms/83 dB or 20 ms/91 dB prepulse with a variable (30, 60, or 120 ms) inter-stimulus interval (ISI: mean = 70 ms), then 40 ms/125 dB pulse; (C) 60 ms no stimulus. Finally, six pulse alone trials were delivered. Habituation was expressed as the percentage reduction in ASR from the initial six baseline trials, to the final six trials. PPI was calculated as follows: [100 − (mean prepulse − pulse trials/mean pulse alone trials) × 100]. Mean ASR was obtained from pulse alone trials. All measures were derived from the average of the area under the curve in arbitrary units (AVG). Animals with a mean ASR (AVG) response lower than 10 were excluded from analyses as non-responders.

### Body Temperature

To evaluate the possible interactive effect of drugs and environmental conditions, we measured rectal temperatures in rats housed singly or in groups of five per cage. In total, 13 measurements were conducted as follows: three drug-free hourly measurements (07:00–09:00 h) followed by administration of (MEPH 5 or 20 mg/kg or VEH) at 09:00 h, then four 30 min measurements (09:30–11:00 h), and finally six hourly measurements (12:00–17:00 h). A digital thermometer was used; each rat was briefly (max. 10 s) immobilized in a Plexiglas tube during the procedure. Rats were kept under controlled laboratory conditions (temperature: 22 ± 2°C, humidity: 30–70%) in the experimental room throughout the study (which was where all temperature measurements were taken).

### Statistics

All statistical analyses were performed using the data analysis software system STATISTICA version 9.1. [StatSoft, Inc. (2010)]. Tests used a default alpha set at *p* = 0.05, two tailed. Behavioral and thermoregulation studies used factorial designs; therefore, analysis of variance (ANOVA) or analysis of covariance (ANCOVA) were used. Where these yielded significant main effects involving a factor with >2 levels or significant interactions, pair-wise *post hoc* comparisons were conducted using Newman–Keuls tests.

#### Behavioral Data (OFT and PPI)

Open field test spatial distribution (thigmotaxis and *T*_center_) and PPI parameters (habituation, ASR, and PPI) were each analyzed using a 2 × 4 factorial ANOVA with testing-onset (5 or 40 min) and drug treatment (VEH or MEPH 2.5, 5, and 20 mg/kg sc.) as between subjects factors. In the case of significant main effects on ASR or habituation, the significant factor was included as a covariate in subsequent analysis of PPI data (using ANCOVA). The temporal pattern of locomotor activity in the OFT (trajectory length in 5 min blocks) was analyzed using a 2 × 4 × 6 mixed factorial ANOVA with testing-onset and drug treatment as between subjects factors, and time blocks (6 × 5 min) as a within-subjects factor.

Additional analyses to compare the potency of nor-MEPH to MEPH were analyzed using one-way ANOVA with five drug treatment levels (VEH or nor-MEPH 5 mg/kg or MEPH 2.5, 5, and 20 mg/kg sc.) as a between-subjects factor. For the OFT, the temporal pattern of locomotor activity was analyzed using a 5 × 6 mixed factorial ANOVA with drug treatment as a between subjects factor and 5 min time blocks as a within subjects factor. Only data from the 5 min testing-onset were used in this analysis (because data for the 40 min testing-onset were not available for all drug treatments).

#### Body Temperature

Data were analyzed using 3 × 2 × 13 mixed factorial design with drug treatment (VEH or MEPH 5 or 20 mg/kg) and home-cage condition (singly- or group housed) as between subjects factors and time (13 measurements) as a within subjects factor.

## Results

### Pharmacokinetics

**Figure 1:**
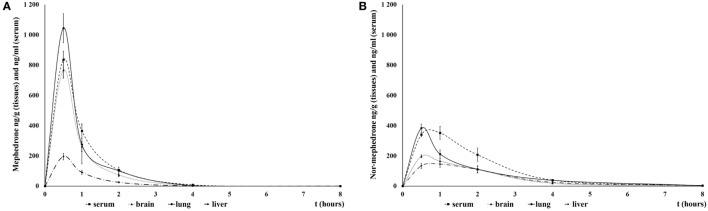
Mean mephedrone (MEPH) **(A)** and its metabolite nor-mephedrone **(B)** levels in serum, brain, lungs, and liver over 6 h after application of MEPH 5 mg/kg sc. Error bars display ±1 SEM.

The maximum mean MEPH serum concentration (826.2 ng/ml) was attained within 30 min. Influx into the brain was not evidently delayed compared to serum; maximum mean concentration in the brain tissue (767 ng/g) was also attained by 30 min after the dose. MEPH robustly accumulated in lung: concentration at 30 min was 1,044.5 ng/g, exceeding concentrations in sera, brain, and liver. Four hours after administration, the levels in sera and all tissues were almost undetectable (Figure 1A).

The maximum mean nor-MEPH (metabolized from MEPH *in vivo*; recall that nor-MEPH itself was not administered in pharmacokinetic studies) serum concentration of 351.9 ng/ml was attained within 1 h of treatment. The maximum mean concentration in the brain (197.1 ng/g) was also evident at 30 min. Nor-MEPH accumulated in lung tissue with a maximum mean concentration of 382.9 ng/g observed at 30 min. Six hours after administration, nor-MEPH was only slightly above the level of detection in all tissues and plasma (Figure 1B).

Mean brain: serum ratio was 1:1.19 for MEPH and 1:1.91 for nor-MEPH throughout the whole temporal observation.

### Behavior

#### Open Field Test

**Figure 2:**
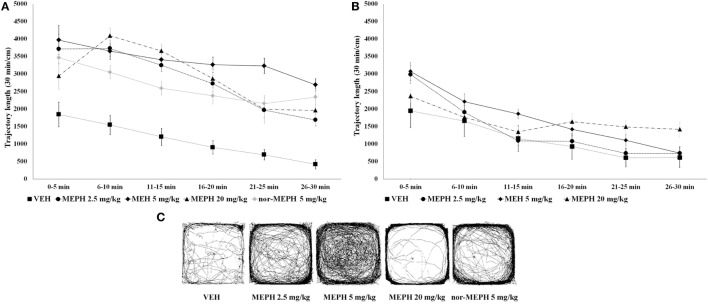
Open field test (OFT): mean trajectory length (divided into 5-min blocks) by testing-onsets [5 and 40 min; **(A)** and **(B)**, respectively] and drug treatments [vehicle controls (VEH), mephedrone (MEPH) 2.5, 5, and 20 mg/kg and nor-mephedrone (nor-MEPH 5 mg/kg)]. Compared to VEH, significant hyperactivity (*p* < 0.001 for all drug groups and in all time blocks) was present at the 5-min testing-onset **(A)**, however the treatment effects were no longer significant at the 40 min testing-onset **(B)**. Error bars display ±1 SEM. Picture inserts below **(C)** show typical trajectory patterns induced by the treatment in animals with 5-min testing-onset.

Analysis of locomotion revealed a main effect of drug treatment [*F* (3, 72) = 24.754, *p* < 0.001], testing-onset [*F* (1, 72) = 72.042, *p* < 0.001] as well as blocks [*F* (5, 360) = 101.67, *p* < 0.001]. All interactions were significant, including the three-way drug × testing-onset × blocks interaction [minimum *F* (15, 360) = 2.979, *p* < 0.001]. The three-way interaction was explored further; at the 5 min testing-onset, while the normal pattern of locomotor habituation (i.e., a progressive decrease in activity over the session) was evident in all groups, *post hoc* tests showed that all MEPH-treated rats were hyperactive (compared to VEH) across the six time blocks (*p* < 0.001) (Figure 2A). At the 40 min testing-onset, elevated activity was no longer present (*p* > 0.05), although rats still showed normal locomotor habituation (Figure 2B). Additional analysis of total locomotion including nor-MEPH (5 min testing-onset) confirmed a significant main effect of drug treatment [*F* (4, 45) = 27.699, *p* < 0.001], blocks [*F* (5, 225) = 50.171, *p* < 0.001], and their interaction [*F* (20, 225) = 3.350, *p* < 0.001]. *Post hoc* tests showed that nor-MEPH 5 mg/kg rats displayed elevated activity (compared to VEH) across all six time blocks (*p* < 0.001) (Figure 2A). For typical trajectory patterns induced by the treatments see Figure 2C.

**Figure 3:**
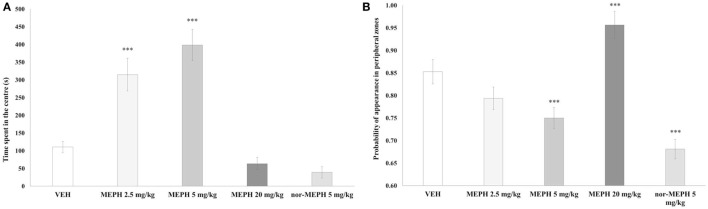
Mean time spent in the arena center [*T*_center_, **(A)**] and mean probability of appearance in peripheral zones [thigmotaxis, **(B)**] after vehicle controls (VEH), mephedrone (MEPH) 2.5, 5, and 20 mg/kg, and nor-mephedrone (nor-MEPH) 5 mg/kg administered at the 5-min testing-onset. MEPH 2.5 and 5 mg/kg-treated rats spent significantly more time in the central zones compared to VEH, and thigmotaxis was decreased by MEPH 5 mg/kg and nor-MEPH 5 mg/kg, and increased by MEPH 20 mg/kg. Error bars display ±1 SEM. ***p < 0.001 compared to VEH.

The effects of drug treatment, testing-onset, and their interaction were each significant for both *T*_center_ [minimum *F* (3, 72) = 5.385, *p* < 0.01] and for thigmotaxis [minimum *F* (3, 72) = 6.792, *p* < 0.001]. Additional one-way ANOVA analyses with nor-MEPH confirmed an effect of drug treatment on *T*_center_ [*F* (4, 45) = 26.845, *p* < 0.001] and thigmotaxis [*F* (4, 45) = 48.704, *p* < 0.001]. *Post hoc* tests showed that the 5-min testing-onset, MEPH 2.5 and 5 mg/kg-treated rats spent more time in the center (*p* < 0.001) compared to VEH. Thigmotaxis was reduced after MEPH 5 mg/kg and nor-MEPH 5 mg/kg (*p* < 0.001), and increased after MEPH 20 mg/kg (*p* < 0.001) (Figures 3A,B). No such significant effects were observed at the 40 min testing-onset (data not shown). Finally, MEPH 5 mg/kg treated rats spent more time in the center (*p* < 0.001) and exhibited lower thigmotaxis (*p* < 0.001) at the 5 min compared to 40 min testing-onset; this pattern was absent in the rest of the groups (data not shown).

#### Prepulse Inhibition

Acoustic startle reaction was not affected by drug treatment or testing-onset, or their interaction [maximum *F* (1, 72) = 3.322, *p* > 0.05; see Table 1]. Analysis of habituation data revealed a main effect of drug treatment [*F* (3, 72) = 3.345, *p* < 0.05]; *post hoc tests* revealed reduced habituation in MEPH 2.5 mg/kg rats compared to VEH (*p* < 0.05); the other MEPH doses did not differ from VEH. There was also a significant main effect of testing-onset [*F* (1, 72) = 6.405, *p* < 0.05] manifested as reduced habituation at the 5 min testing-onset compared to 40 min. The drug treatment × testing-onset interaction was not significant.

**Table 1:** Mean values of acoustic startle reaction (ASR) amplitude and percentage of prepulse inhibition (PPI) after vehicle controls (VEH), mephedrone (MEPH), and nor-mephedrone (nor-MEPH) by testing-onsets (5 and 40 min).

		Drug treatment				
Measure	Testing-onsets (min)	VEH	MEPH 2.5 mg/kg	MEPH 5 mg/kg	MEPH 20 mg/kg	nor-MEPH 5 mg/kg
ASR	5	104.5 (14.3)	117.5 (17.4)	155.5 (32.7)	110.5 (14.2)	72.1 (11.5)
	40	137.2 (20.0)	140.8 (26.4)	144.6 (22.3)	173.9 (24.8)	–
% PPI	5	36.8 (5.4)	32.8 (5.6)	31.1 (6.2)	31.3 (4.1)	30.2 (6.5)
	40	41.3 (3.7)	41.1 (2.1)	25.1 (7.5)	28.4 (3.3)	–

*Numbers represent means and SEMs are shown in brackets. Differences between testing-onsets and drug treatments were non-significant*.

Since there were significant effects of drug treatment and testing-onset on habituation, it was included as a covariate in PPI analyses. PPI was not affected by the drug treatment or testing-onset, while their interaction was significant [*F* (3, 71) = 3.483, *p* < 0.05]. At the 40 min testing-onset, means suggested some disruption of PPI (MEPH 5 and 20 mg/kg); however, *post hoc tests* comparisons showed that differences from VEH were only marginal (*p* = 0.062, *p* = 0.081, respectively). There were no clear differences in means (eye-balling the data) at 5 min that seemed likely to account for the significant interaction; since a further one-way ANOVA was planned to explore effects of MEPH (alongside nor-MEPH) on PPI, further *post-hoc* tests on the 5 min testing-onset data were not conducted at this time. This additional one-way ANOVA showed no significant effect of treatment (MEPH or nor-MEPH) on PPI at the 5 min testing-onset [*F* (4, 45) = 0.696, *p* > 0.05]; therefore, the marginal effects at 40 min must explain the previous interaction. Similarly, there was no effect of MEPH or nor-MEPH on ASR [*F* (4, 45) = 2.454, *p* > 0.05] or habituation [*F* (4, 45) = 1.912, *p* > 0.05] at the 5-min testing-onset.

### Body Temperature

**Figure 4:**
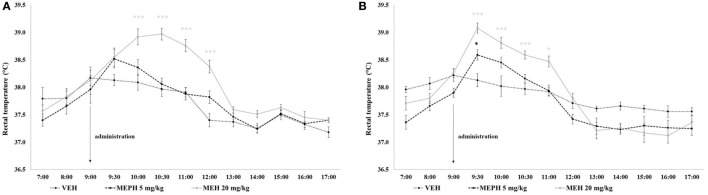
Mean rectal temperature (°C) over 10 h after vehicle control (VEH), and mephedrone (MEPH) 5 and 20 mg/kg treatments for rats housed singly **(A)** or in groups of five **(B)**. Substances were administered at 09:00 h. Temperatures of rats treated by 5 mg/kg did not differ from VEH, except for the short-term elevation in the first 30 min after the administration in group-housed rats. The increase induced by 20 kg/kg was maintained from 10:00 to 12:00 h in singly housed rats and from 09:30 to 11:00 h in group-housed rats. Error bars display ±1 SEM. *p < 0.05, ***p < 0.001 compared to VEH.

Rectal temperature was significantly affected by drug treatment [*F* (2, 54) = 9.409, *p* < 0.001] and time [*F* (12, 648) = 124.560, *p* < 0.001] but not home-cage condition [*F* (1, 54) = 0.127, *p* > 0.05]. All interactions were significant including the three-way drug treatment × time × home-cage interaction [minimum *F* (12, 648) = 2.406, *p* < 0.010]. *Post-hoc* tests revealed no significant differences between MEPH 5 mg/kg and VEH groups, except the elevation (~0.5°C) which occurred in the first 30 min after administration in group-housed rats (*p* < 0.05). Compared to VEH, MEPH 20 mg/kg induced modest elevation (~0.4°C) in singly-housed rats that appeared in the first 30 min after administration; however, it became statistically significant 30 min later and the effect was maintained for the next 2 h (~1°C; minimum *p* < 0.001). In group-housed rats, the elevation became significant within first 30 min and remained increased for next 2 h (~1°C; minimum *p* < 0.001)—Figure 4.

## Discussion

Mephedrone quickly peaked in the serum and was rapidly incorporated into all tissues, with lungs showing the highest concentrations and liver the lowest. MEPH was almost undetectable in serum and tissue by 4 h after its administration. Nor-MEPH had a similar profile; however the concentrations of nor-MEPH decreased more gradually in comparison to the parent drug (with MEPH, a steep decrement occurred immediately after the peak). Therefore, compared to MEPH, the elimination of nor-MEPH was slightly delayed. Acute administration of both compounds resulted in dose-dependent stimulatory effects, disrupted habituation, and altered the spatial distribution of locomotor behavior in the open field; however, there was no significant effect on PPI. MEPH induced dose- and environment-dependent increases in rectal temperature (of up to ~1°C) in both group-housed rats (as expected), but also in singly housed rats, where temperature remained elevated for 3 h after administration of the highest MEPH dose.

### Pharmacokinetics

In their study with iv. administration, Aarde et al. (29) showed that MEPH peaked in the brain within 2 min; since the most pronounced locomotor effects in our study were present within 5–10 min of administration, it is likely that the peak concentration in serum also occurred earlier than suggested by our pharmacokinetic study (where the first measurement was at 30 min after the sc. administration). As expected, we detected the highest serum levels of both compounds in our dataset slightly earlier compared to oral administration, where MEPH peaked in serum within 45 min–1.5 h after administration (48). The speed of crossing the blood–brain barrier by MEPH implied by our current results was consistent with Aarde et al. (29); as shown by others (20), MEPH easily crosses blood–brain barrier and, thus, influx into brain and lung tissues is most likely due to its lipophilic profile. This finding is also consistent with the pharmacokinetics of another ring-substituted cathinone, methylone (39) as well as with the phenethylamines 2C-B and PMMA, aminoindanes such as MDAI where highest tissue concentrations were detected in lungs and brains (41, 49, 50). Not surprisingly, since nor-MEPH is not the only one major metabolite, it reached lower overall serum and tissue levels than the parent drug and the slope of its elimination was less steep, resulting in higher serum and brain concentrations compared to MEPH 3 h after its administration. One possible explanation could be the slightly higher polarity of nor-MEPH leading to slower crossing of the blood–brain barrier (30) and, theoretically, nor-MEPH may, therefore, be responsible for some delayed or prolonged effects of MEPH.

### Behavioral Effects: Open Field and PPI

In line with pharmacokinetics, locomotor stimulant effects declined quickly, so MEPH and nor-MEPH lacked any significant stimulatory effects 40 min after administration. The rapid action of MEPH observed here is in line with other rodent studies (23, 28, 30) and reports from human users (10). Since MEPH and nor-MEPH have both been shown to act on DAT (23, 30), it is most likely the underlying cause of these effects (51). MEPH and nor-MEPH seemed to be behaviorally equipotent. The fact that the effects lasted a very short time (due to fast kinetics) may increase the likelihood of re-dosing by humans and, together with its strongly reinforcing effects (shown in self-administration studies), indicates highly addictive characteristics (10).

Spatial characteristics of the trajectory after MEPH showed bi-directional effects dependent on the dose used. While increased exploration of the central zones following lower doses might imply decreased anxiety, increased thigmotaxis following the highest dose could suggest the opposite (52, 53). Compared to our findings, studies measuring anxiety using the elevated plus-maze (EPM) revealed contradictory results including either increased anxiety after acute treatment with low doses [0.25–10 mg/kg (54)], or no effect after sub-chronic MEPH treatment with very high doses (30 mg/kg twice a day) (55, 56). Direct comparison of anxiety measures in the OFT versus EPM, however, may be difficult. While some authors report a good comparability (57) others have questioned this (58). In our study, spatial trajectory characteristics may be also affected by other mechanisms, such as increased stereotyped behaviors (e.g., circling the perimeter of the arena) such as was also observed in our previous studies with other related compounds (38, 41, 47).

In accordance with previous research (31), we did not see any significant effect of acute MEPH or nor-MEPH on PPI. When our data are compared with similar data sets from phenethylamines, cathinones and aminoindanes performed in our laboratory, it is evident that that the more serotonergic the drug is [e.g., according to their DAT: SERT inhibition ratios (20)], the more pronounced the disruptive effect on PPI. While MDMA, PMMA, and MDAI significantly disrupted PPI at the lowest doses used (35, 38, 41), which have mild-to-moderate stimulatory effects and do not induce stereotyped circling in the OFT, amphetamine and MDPV was effective only at the highest dose used where stereotyped behaviors were also evident [(37); unpublished observation Horsley et al.]. MEPH has also shown some activity at 5-HT_2A_ receptor (20), however, it is not clear whether it acts as agonist or antagonist. In relation to this, disruption of PPI is typically seen after administration of various 5-HT_2A_ agonists, serotonergic hallucinogens, such as LSD, mescaline, psilocybin, 2C-B or DOI, etc., and it is known that antagonists at this receptor can reinstate normal PPI (37, 59–63). Similarly, MDMA-induced PPI deficits in rats can be also normalized by 5-HT2A antagonists (64, 65), therefore suggesting a role for this receptor subtype in PPI; if MEPH acts as an antagonist at 5-HT2A receptors, this might theoretically be protective against psychomimesis.

### Temperature

The hypothesis that MEPH, such as other cathinones (7), has a potency to alter thermoregulation was supported by evidence in our study. It is in line with reports of recreational users suffering from adverse effects related to altered peripheral thermoregulation, such as cold-blue fingers, hot flushes, and/or intensive sweating (9, 26). Likewise comparable preclinical studies [for review, see Green et al. (7)], we observed significant hyperthermia in both singly housed as well as group-housed rats under normal room temperature (22 ± 2°C). In contrast to our expectations, the temperature increase was almost identical (~1°C) in both groups but had slightly longer duration in singly housed rats. A possible explanation might be the faster onset of the temperature increase in the group-housed animals, where aggregation of animals in one cage would increase the microclimate temperature and in turn increase the speed of metabolism. The persistence of the temperature increase (3 h in singly housed rats), surprisingly, did not correspond with the rapid pharmacokinetic and locomotor profile of MEPH. Therefore additional factors, such as other active metabolite/s, may contribute to this prolonged effect and may indicate a potential for prolonged somatic drug toxicity, as in the case of toxic MDMA metabolites (66). In general, thermoregulation is mainly affected by drugs that primarily target serotonergic system [e.g., MDMA, PMMA, or MDAI (38, 41, 67)]. Dopaminergic stimulants may also increase body temperature (by increasing the behavioral activity), but effects are not as robust as with serotonergics (7). Direct comparisons of MEPH with other related cathinones, methylone 20 mg/kg sc., and MDPV 2 mg/kg sc. tested in our laboratory shows that the temperature increase was similar [(39); unpublished observation Horsley et al.]. This is of interest since the stimulant activity relative to the potency of the drug (DAT inhibition) should be approximately the same; however, the inhibition of SERT is much lower compared to DAT, and in the case of the lower MPDV dose would be approximately five times less effective (inhibiting SERT) than with MEPH or methylone (20). Taken together with the fact that the temperature increase was more prolonged in singly- than in group-housed rats and that it did not exceed 40°C, we suggest that increases in the overall behavioral activity relevant to dopaminergic stimulation are responsible for the hyperthermia observed. However, against this interpretation, locomotor activation disappeared within 40 min of administration which is not consistent with the prolonged temperature increases. Further experiments will be needed in order to explain these discrepancies.

## Conclusion

To conclude, both MEPH and nor-MEPH had rapid kinetics with accumulation in lungs and behaved as short-acting, potent stimulants with low capacity to disrupt sensorimotor gating. Dissociation between the duration of behavioral and hyperthermic effects may be due to the presence of another active metabolite with slower pharmacokinetic profile and may be indicative of prolonged risk of somatic toxicity even though acute stimulant-like effects have already worn off.

## Ethics Statement

All procedures were conducted in accordance with the principles of laboratory animal care of the National Committee for the Care and Use of Laboratory Animals (Czech Republic), and according to Guidelines of the European Union (86/609/EU). The protocol was approved by the National Committee for the Care and Use of Laboratory Animals (Czech Republic) under the number: MEYSCR-27527/2012-31.

## Author Contributions

All authors made a substantial contribution to the conception or design of the work; or the acquisition, analysis, or interpretation of data for the work. All authors were involved in drafting the work or revising it critically for important intellectual contents. All authors gave final approval for the current version of the work to be published. All authors agree to be accountable for all aspects of the work in ensuring that questions related to the accuracy or integrity of any part of the work are appropriately investigated and resolved.

## Conflict of Interest Statement

The authors declare that the research was conducted in the absence of any commercial or financial relationships that could be construed as a potential conflict of interest.
